# The use of lipid-lowering therapy and effects of antihyperglycaemic therapy on lipids in subjects with type 2 diabetes with or without cardiovascular disease: a pooled analysis of data from eleven randomized trials with insulin glargine 100 U/mL

**DOI:** 10.1186/s12933-017-0548-0

**Published:** 2017-05-19

**Authors:** Markolf Hanefeld, Louise Traylor, Ling Gao, Wolfgang Landgraf

**Affiliations:** 1 Study Center Metabolic Vascular Medicine, GWT-TU Dresden GmbH/UKD, Medical clinic III, Fiedler Str. 34, 01307 Dresden, Germany; 2 University Hospital Carl Gustav Carus, Dresden, Germany; 3 Sanofi Us Inc., 55 Corporate Dr, Bridgewater, NJ 08807 USA; 4 Analysta Inc., Somerset, NJ USA; 5 Sanofi, K703, Industriepark Höchst, 65926 Frankfurt Am Main, Germany

**Keywords:** Type 2 diabetes, Cardiovascular disease, Lipid control, Statins, Clinical trials

## Abstract

**Background:**

Dyslipidaemia is a major contributor to the increased risk of cardiovascular disease (CVD) associated with type 2 diabetes (T2D). This study aimed to characterize the extent of lipid-lowering therapy use and its impact on lipid and glycaemic outcomes in people with T2D uncontrolled on oral agents who were enrolled in insulin glargine 100 units/mL (Gla-100) randomized controlled trials (RCTs).

**Methods:**

A post hoc patient-level pooled analysis of eleven RCTs (≥24 weeks’ duration) comparing Gla-100 (±oral antidiabetes drugs [OADs]) with OADs alone in people with T2D was performed. Baseline and Week 24 or study endpoint lipid status (low-density lipoprotein cholesterol [LDL-C], high-density lipoprotein cholesterol [HDL-C], non-high-density lipoprotein cholesterol [non-HDL-C] and triglycerides) and indices of glycaemic control (glycosylated haemoglobin, fasting plasma glucose [FPG]) were examined in patient groups according to treatment received and CVD status. Lipid-lowering therapy was provided at the discretion of physicians at baseline and throughout the studies.

**Results:**

Of the 4768 participants included in the analysis, 41% (n = 1940) received lipid-lowering therapy. Only 51% of participants with CVD (1885/3672) were treated with lipid-lowering therapy; these participants had significantly lower levels of LDL-C, HDL-C and non-HDL-C, and higher levels of triglycerides versus patients not treated with lipid-lowering therapy at baseline and study endpoint (*P* < 0.001 for all). Antihyperglycaemia therapy resulted in decreases in glycosylated haemoglobin (−1.4 to −1.6%) and FPG (−68.9 to −75.3 mg/dL) at Week 24. Furthermore, slight improvements in non-HDL-C (−3.9 to −9.1 mg/dL) and triglyceride levels (−25.8 to −51.2 mg/dL) were observed. Similar changes were seen irrespective of lipid-lowering therapy or CVD status.

**Conclusions:**

In a T2D cohort included in Gla-100 clinical studies, many participants with T2D and CVD did not receive lipid-lowering therapy, and for most categories of lipid the levels were outside the optimal range. Even in patients treated with antihyperglycaemic therapy but not lipid-lowering therapy, there were modest improvements in non-HDL-C and triglyceride levels in all participants with T2D and CVD. There is a need for increased implementation of guideline recommendations such as American College of Cardiology/American Heart Association for the management of dyslipidaemia in patients with T2D.

## Background

Dyslipidaemia is recognized as a major risk factor for cardiovascular disease (CVD), which is a significant cause of morbidity and mortality in type 2 diabetes (T2D) [1–3]. Reducing hyperglycaemia in patients with diabetes has been shown to decrease onset and progression of microvascular complications, although the impact on cardiovascular complications varies depending on individual risk, quality of overall risk factor control and antidiabetes drugs used [3–10]. The use of antidiabetes therapies (including insulin and oral antidiabetes drugs) has been shown to affect lipid levels [11–14]. This is often related to a therapy’s interaction with lipid metabolism, which can result in varied effects on cardiovascular complications. In this complex network of interactions, low-density lipoprotein cholesterol (LDL-C) is a key player as a coronary risk factor. Data from the 2005–2008 National Health and Nutrition Examination Survey (NHANES) indicate that more than one-third (33.5%) of adults in the US age ≥20 years—equivalent to 71 million people—have elevated levels of LDL-C [15]. However, less than half of this number (48.1%) receives treatment, and only 33.2% have their cholesterol under control [16]. Increased LDL-C is the most recognized form of dyslipidaemia; however, high levels of triglycerides and low levels of high-density lipoprotein cholesterol (HDL-C) may also be harmful [17], particularly in T2D.

Diabetes is commonly associated with a phenotype of mixed dyslipidaemia. This is characterized by low HDL-C and high triglyceride levels, with often normal or modestly elevated LDL-C levels [1]. However, normal LDL-C levels in diabetes may be misleading, as there is generally an increase in the number of small, dense atherogenic LDL and cholesterol-enriched remnant particles [1, 18]. Therefore, in patients with diabetes, non-HDL cholesterol may be a stronger predictor of CVD than LDL cholesterol or triglycerides, as levels correlate highly with atherogenic lipoproteins [18].

Until recently, the majority of guidelines for cholesterol treatment and CVD prevention have focused on targets for lipids, broadly set at: <100 mg/dL for LDL-C; <150 mg/dL for triglycerides; and >50 mg/dL for HDL-C [17]. However, as in the majority of branches of medicine, up-to-date guidelines focus on a personalized approach rather than a blanket recommendation for all patients. Current American College of Cardiology/American Heart Association (ACC/AHA) guidelines recommend the use of a risk-assessment algorithm to calculate 10-year risk of CVD [19, 20]. The cholesterol-treatment guideline recommends an algorithm which takes into account a range of factors when deciding on the need for lipid-lowering therapy. These include age, race, smoking presence of CVD or history of major cardiovascular events and diabetes status, blood pressure, LDL and cholesterol levels [19, 21]. Current recommendations for the general population suggest that statins should be introduced if the 10-year risk of CVD is greater than 7.5% [19]. For patients with diabetes, statin use is recommended for all patients between the ages of 40 and 75 years and with LDL-C 70–189 mg/dL. High intensity statin use is recommended for patients with diabetes and 10-year risk of CVD above 7.5% or with CVD [19, 22]. Reflecting this, recent outcome studies investigating intensified glucose control require control of major risk factors (e.g. lipids, blood pressure) as a precondition. However, despite detailed guidelines, data indicate that the majority of patients at high risk of CVD, including those with diabetes, are failing to attain lipid-goals [15, 23, 24].

Although reducing hyperglycaemia has been shown to decrease onset and progression of microvascular complications, the impact on CV complications is unclear. The aim of this patient-level pooled analysis was to characterize the extent of lipid-lowering therapy use and the degree of cholesterol control in patients with T2D with and without CVD enrolled in head-to-head randomized trials, together with lipid and glycaemic outcomes following initiation of antihyperglycaemia therapy.

## Methods

### Study and patient selection

Eligible studies were randomized controlled trials conducted using insulin glargine 100 units/mL (Gla-100). Trials investigated Gla-100 used alone or in combination with other agents versus comparators, or compared treatment initiation support methods for Gla-100. Therapies were titrated to a fasting plasma glucose (FPG) target of ≤100 mg/dL (≤5.6 mmol/L) for duration of ≥24 weeks. Lipid measurements were available for all included trials. Lipid-lowering therapy was provided at the discretion of participating physicians at baseline and throughout the duration of the studies. Baseline and Week 24/endpoint lipid status and indices of glycaemic control were examined in the various patient groups according to lipid-lowering treatment received and presence of risk factors. Five main subpopulations were investigated: all participants who received lipid-lowering therapy; all participants who did not receive lipid-lowering therapy; participants diagnosed with CVD at baseline and who received lipid-lowering therapy; participants with CVD who did not receive any lipid-lowering therapy; participants without CVD and who did not receive lipid-lowering therapy.

#### Target parameters

The following parameters were analysed in participants with both baseline and endpoint values available:

- Glycosylated haemoglobin (HbA1c) and FPG.
  - Assessed at baseline and endpoint/Week 24.
- Lipid parameters, including low-density lipoprotein cholesterol (LDL-C), high-density lipoprotein cholesterol (HDL-C), non-high-density lipoprotein cholesterol (non-HDL-C) and triglycerides.
  - Assessed at baseline and endpoint/Week 24.

#### Statistical analyses

The pooled analysis was performed by lipid-lowering therapy categories (treatment vs. no treatment) and CVD groups based on standardized patient-level data generated from the identified studies. Demographic and baseline clinical characteristics of age, gender, weight, body mass index (BMI), duration of diabetes, HbA1c and FPG were summarized by lipid-lowering therapy categories and CVD groups. They were also examined for lipid-lowering therapy categories and CVD groups using t-tests for continual variables and Chi square test for gender. HbA1c and FPG at baseline and Week 24 were reported descriptively and compared between lipid-lowering therapy categories and CVD groups using analysis of covariance (ANCOVA) models. These included age, duration of diabetes, baseline BMI, HbA1c and FPG as covariates. They also used gender, lipid-lowering therapy category (treatment vs. no treatment), CVD group, study and randomized treatment arm for type of insulin or oral antihyperglycaemia drug group as fixed factors. Lipid variables at baseline and Week 24 were reported descriptively and compared between lipid-lowering therapy categories and CVD groups using ANCOVA models. These included age, duration of diabetes, baseline BMI, HbA1c, FPG and the corresponding baseline lipid value as covariates. They also used gender, lipid-lowering therapy category (treatment vs. no treatment), CVD group, study, and randomized treatment arm for type of insulin or oral antihyperglycaemia drug group as factors. The lipid data were log transformed to be more normal prior to the model estimation; adjusted means have been antilogged and presented in the original scale as the geometric means.

## Results

### Eligible studies

A total of 11 studies conducted from 1999 to 2008 fulfilled the inclusion criteria (Table 1).

**Table 1:** Summary of included studies

Study	Phase	Treatment	Number of subjects randomized/ treated	Treatment period, weeks	Insulin titration schedule
EASIE [35]	3b/4	Gla-100 + MET vs. SITA + MET	515/501	24	Twice weekly
4020 [36]	3b	Gla-100 + SU or MET vs. PIO + SU or MET	389/382	24 extended to 48	Weekly
4022 [37]	3b	Gla-100 + SU or MET vs. TZD + SU + MET	337/334	24 extended to 48	Weekly
L2T3 [38]	4	Gla-100 + OADs vs. DET + OADs	973/964	24	Every 2 days
IN-SIGHT [39]	3b	Gla-100 + current OADs vs. current OADs	405/400	24	Daily
4001 [40]	3b	Morning vs. bedtime Gla-100 + morning GLIM vs. NPH insulin bedtime + morning GLIM	700/697	24	Weekly
4013 [41]	3b	Gla-100 bedtime + morning GLIM vs. NPH insulin bedtime + morning GLIM	528/481	24	Weekly
4002 [42]	3b	Gla-100 bedtime + OADs vs. NPH insulin bedtime + OADs	764/756	24	Weekly
4014 [43]	4	Gla-100 + SU + MET vs. ROS + SU + MET	219/217	24	Weekly
4021 [44]	3b	Gla-100 + SU + MET vs. LIS 75/25 + SU + MET	212/212	24	Weekly
4041 [45]	4	Gla-100 with group education + OADs vs. Gla-100 with individual education + OADs	121/121	24	Self-titration, then investigator reviewed at each visit

*DET* insulin detemir, *GLIM* glimepiride, *LIS* insulin lispro, *MET* metformin, *OAD* oral antidiabetes drug, *PIO* pioglitazone, *ROS* rosiglitazone, *SITA* sitagliptin, *SU* sulfonylurea, *TZD* thiazolidinedione

### Patient characteristics

In total, data from 4768 participants were analysed, of whom 41% (n = 1940) received lipid-lowering therapy. Of the total number of participants, 40% (n = 1885) had diagnosed CVD at baseline and received lipid-lowering therapy at the discretion of their physicians throughout the 6-month study duration. Also, of the total number of participants 37% (n = 1787) had CVD and did not receive any lipid-lowering therapy during the study period. Furthermore, 22% (n = 1041) of the total number of participants did not have CVD and did not receive lipid-lowering therapy during the study period (Table 2). Overall, only 51% (1885/3672) of the total number of study participants with CVD were treated with lipid-lowering therapy. Key baseline characteristics by lipid-lowering treatment status are presented in Table 2. There were significant differences between treated and untreated patients across all characteristics examined (Table 2). Compared with untreated patients, treated patients were older, more likely to be male, and had a greater body weight, longer duration of diabetes and higher fasting C-peptide level (*P* < 0.001 for all). Compared with untreated patients without CVD, untreated patients with CVD were more likely to be female, have a longer duration of diabetes, weigh more and have a higher BMI (*P* < 0.01 for all).

**Table 2:** Patient demographics and baseline characteristics

Characteristic	Lipid-lowering drug treatment		No lipid-lowering drug treatment			*P* value
	Total (n = 1940)	With CVD (n = 1885)	Total (n = 2828)	With CVD (n = 1787)	Without CVD (n = 1041)	Treated vs non-treated (total populations)
Male, %	58	58	50	48*	53	<0.001
Age, years	57.9 (9.0)	57.9 (9.0)	55.7 (10.1)	57.1* (9.7)	53.1 (10.4)	<0.001
Weight, kg	89.4 (19.1)	89.6 (19.1)	86.5 (21.2)	88.5* (20.3)	83.3 (22.2)	<0.001
BMI, kg/m^2^	31.4 (5.4)	31.5 (5.4)	30.9 (6.1)	31.7* (5.8)	29.6 (6.5)	0.004
T2D duration, years	9.1 (6.3)	9.1 (6.3)	8.5 (5.8)	8.7* (5.9)	8.0 (5.5)	<0.001
HbA1c, %	8.75 (1.03)	8.75 (1.03)	8.89 (1.08)	8.84* (1.06)	8.98 (1.11)	<0.001
FPG, mg/dL	196 (56)	196 (55)	200 (57)	200 (57)	201 (58)	0.01
FPG, mmol/L	10.9 (3.1)	10.9 (3.1)	11.1 (3.2)	11.1 (3.2)	11.2 (3.2)	0.01
Fasting C-peptide, nmol/L	1.18 (0.59)	1.19 (0.60)	1.10 (0.60)	1.15 (0.59)	1.01 (0.60)	<0.001

Data presented represent mean (standard deviation) unless otherwise specified

*HbA1c* glycosylated haemoglobin, *BMI* body mass index, *CVD* cardiovascular disease, *T2D* type 2 diabetes, *FPG* fasting plasma glucose

* Statistically significant differences (P < 0.01) between with-CVD and without-CVD groups

Lipid-lowering therapy included statins (n = 1751; 88%), fibrates (n = 218; 11%), and other agents (n = 185; 10%). The participants with CVD who were treated with lipid-lowering therapy group (n = 1885) consisted of: 51% (n = 964) receiving Gla-100 as glucose-lowering therapy, 16% (n = 299) receiving other insulins (insulin detemir or premixed insulin), 14% (n = 263) receiving NPH insulin, and 19% (n = 359) receiving oral antihyperglycaemia drugs only. The participants with CVD at baseline who were not treated with lipid-lowering therapy group (n = 1787) consisted of: 53% (n = 950) receiving Gla-100, 10% (n = 176) receiving other insulins, 17% (n = 303) receiving NPH insulin, and 20% (n = 358) receiving oral antihyperglycaemia drugs. The participants without CVD at baseline who were not treated with lipid-lowering therapy group (n = 1041) consisted of: 54% (n = 564) receiving Gla-100, 7% (n = 69) receiving other insulins, 24% (n = 248) receiving NPH insulin, and 15% (n = 160) receiving oral antihyperglycaemia drugs.

### Glycaemic control

**Fig. 1:**
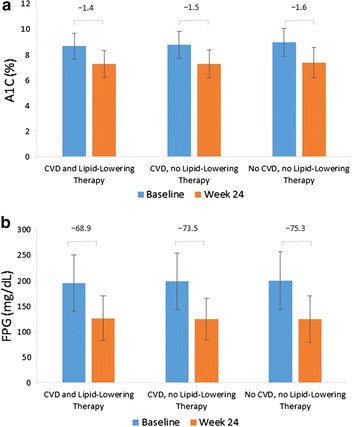
HbA1c (**a**) and FPG (**b**) at baseline and Week 24 ±CVD or lipid-lowering therapy. Data presented represent mean (standard deviation). *P* < 0.05 between patients treated with lipid-lowering therapy versus not treated for HbA1c at baseline and change to Week 24, differences were not significant in FPG

Overall, participants who received lipid-lowering therapy had a lower baseline HbA1c than those who did not (8.75% vs. 8.89%, respectively; *P* < 0.001). However, they had a higher adjusted HbA1c at endpoint (7.34% vs. 7.22%; *P* < 0.0001) and a smaller change from baseline to endpoint (−1.50% vs. −1.61%; *P* < 0.0001). In terms of CVD status and lipid-lowering therapy, HbA1c was reduced from baseline to Week 24 similarly across all three patient subgroups (Fig. 1a).

Participants who received lipid-lowering therapy had a lower baseline FPG than those who did not (196 mg/dL vs. 200 mg/dL, respectively; *P* = 0.01). There was no statistical difference in adjusted endpoint FPG (128.8 mg/dL vs. 127.6 mg/dL; *P* = 0.2222) or change from baseline to adjusted endpoint FPG (−69.7 mg/dL vs. −70.9 mg/dL; *P* = 0.2222). When stratified by CVD status and lipid-lowering therapy, FPG was reduced across the three examined groups from baseline to Week 24 (Fig. 1b). Reductions were marginally greater in participants who did not receive lipid-lowering therapy, regardless of their CVD status.

### Lipid status

**Fig. 2:**
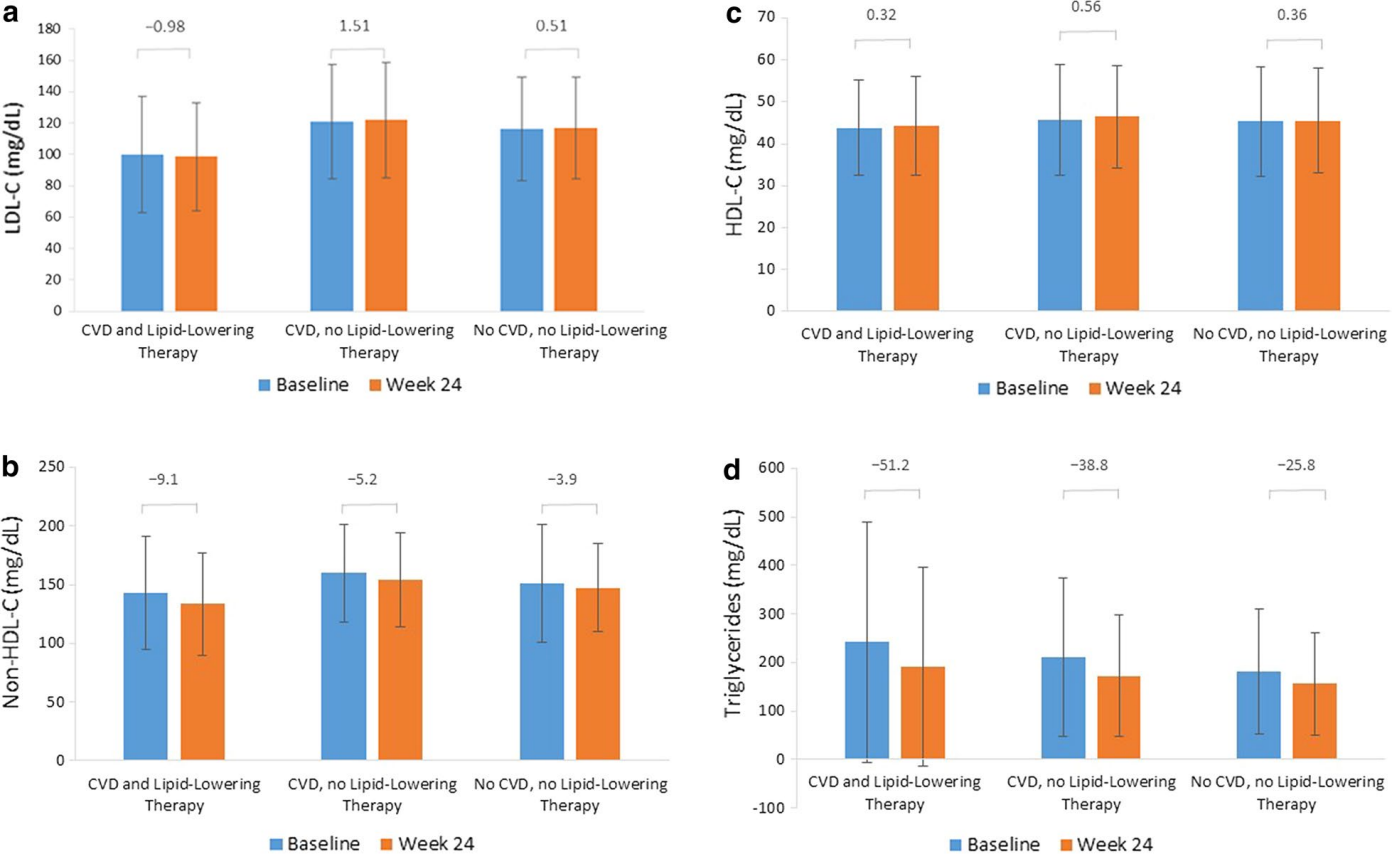
Lipid status at baseline and Week 24 ±CVD or lipid-lowering therapy: LDL-C (**a**), Non-HDL-C (**b**), HDL-C (**c**), and triglycerides (**d**). Data presented represent mean (standard deviation). *P* < 0.05 between patients treated with lipid-lowering therapy versus not treated for all lipid parameters at baseline and change to Week 24, except for triglycerides which were significant (*P* < 0.05) at baseline only

At baseline, participants treated with lipid-lowering therapy (compared with untreated patients) had lower levels of LDL-C (99.8 mg/dL vs. 119.2 mg/dL, respectively; *P* < 0.001), non-HDL-C (142.6 mg/dL vs. 156.6 mg/dL; *P* < 0.001) and HDL-C (43.9 mg/dL vs. 45.5 mg/dL; *P* < 0.001), and higher levels of triglycerides (240.6 mg/dL vs. 200.3 mg/dL; *P* < 0.001). Results were similar at Week 24 for LDL-C (98.5 mg/dL vs. 120.1 mg/dL; *P* < 0.001), non-HDL-C (133.4 mg/dL vs. 151.6 mg/dL; *P* < 0.001), HDL-C (44.2 mg/dL vs. 46.0 mg/dL; *P* < 0.036), and triglycerides (189.2 mg/dL vs. 166.0 mg/dL; *P* = 0.1223). In the groups stratified by CVD status and lipid-lowering therapy, non-HDL-C and triglyceride levels slightly improved, while LDL-C and HDL-C levels remained almost unchanged following therapy with antihyperglycaemia drugs, irrespective of receipt of lipid-lowering therapy for these groups (Fig. 2a–d).

## Discussion

In this pooled analysis of head-to-head randomized trials of Gla-100 in T2D, approximately 41% of all participants were treated with lipid-lowering therapy. Of those patients with CVD at baseline, only 51% of participants received lipid-lowering therapy. Following initiation of antihyperglycaemia treatment, HbA1c was reduced from baseline to study endpoint in participants with CVD—both with and without lipid-lowering therapy—and in untreated participants without CVD. Those participants receiving lipid-lowering therapy had a lower baseline HbA1c, and achieved smaller reductions in HbA1c from baseline to endpoint. Baseline FPG was lower in lipid-lowering therapy-treated participants and reduced in all CVD/treatment groups examined from baseline to endpoint. In terms of lipid profiles, all forms of cholesterol were lower in participants treated with lipid-lowering therapy at baseline, while triglyceride levels were higher; this pattern persisted to Week 24. There were reductions in triglyceride levels from baseline to Week 24 in all CVD/lipid-lowering treatment groups, as well as slight reductions in non-HDL-C; LDL-C and HDL-C levels were not substantially changed during therapy.

There were significant differences in baseline characteristics between treated and untreated participants in this analysis. These were likely driven by the higher number of patients with CVD in the treated participants group, given that CVD is more common in males, increases with duration of diabetes, and is associated with elevated serum C-peptide [25, 26]. We also found significant differences in baseline characteristics between untreated patients with and without CVD. The higher proportion of females in the untreated CVD group may be driven by under-recognition of CVD in women leading to later and less aggressive treatment. This may also have contributed to the fact that patients in this group were older and had a longer duration of diabetes [27, 28].

Although there were significant differences in baseline glycaemic control for participants who were treated with lipid-lowering therapy versus those who were not, these differences were numerically small (0.14%), and their clinical relevance is questionable. The slight advantages in glycaemic control at baseline in those participants treated with lipid-lowering therapy may be indicative of a more stringent prior treatment regimen or greater patient engagement with healthcare goals prior to trial entry. This may also account for the use of lipid-lowering therapy in these participants. Overall, all three CVD/treatment groups showed a reduction in HbA1c of around 1.5%. The relevance of the difference in HbA1c change between treated and untreated participants, although significant, is slight (0.11%) and unlikely to be clinically relevant. Reductions in HbA1c can be considered a positive outcome in relation to CVD, as elevated HbA1c has been shown to have a strong correlation with increased CVD risk in patients with diabetes [29, 30].

There is a strong evidence base for statin therapy in all patients with diabetes and between the ages of 40 and 75 years [19, 22]. However in our population, which had a median age of 56.5 years, less than half of all participants were receiving lipid-lowering therapy. Of notice, AHA and ESC guidelines recommend that all patients with clinical CVD receive lipid-lowering therapy, regardless of their diabetes status [19], with current guidelines indicating the need for high intensity statin therapy for all patients with CVD and T2D [22]. Despite this, only 51% of participants with CVD and diabetes in our study received lipid-lowering therapy.

Reducing LDL-C is the major focus of CVD prevention. Under current ACC/AHA guidelines, which concentrate on statin treatment based on a CVD risk-assessment algorithm, there is no longer a LDL-C goal [19, 22]. However, previous guidelines (1998–2008) set <100 mg/dL as the optimal LDL-C level in high-risk groups, indicating that participants treated with lipid-lowering therapy in our study had, according to current guidelines, optimal LDL-C at baseline and Week 24, while those without treatment were above this optimal goal regardless of CVD status [17]. Elevated triglycerides may be overlooked in patients with well controlled LDL-C [1, 31], and baseline triglycerides were high (>200 mg/dL) in both treated and untreated participants in our study [17]. Following therapy with antihyperglycaemia agents, triglyceride levels fell to within the ‘borderline high’ category (150–199 mg/dL) for both treated and untreated participants. This is in keeping with previous reports of reductions in triglycerides being associated with antihyperglycaemia treatment, and with the use of insulin therapy in cases of severely elevated triglycerides [11–14]. The fact that this has been seen with treatments other than insulin suggests that this is the result of reduced glycaemia rather than being a direct effect of the treatment.

As noted previously, mixed dyslipidaemia (high triglycerides/low HDL-C) is common in patients with diabetes [1]. This pattern was observed in our study population, with HDL-C levels being consistently below the optimal level of 50 mg/dL [17]. In high-risk individuals with controlled LDL-C but high triglyceride levels, a non-HDL-C target of <130 mg/dL has been recommended [18], although guidelines indicate that there is insufficient evidence from randomized controlled trials to determine either LDL-C or non-HDL-C treatment targets [19]. In our study, mean non-HDL-C was above the <130 mg/dL target in both treatment categories at baseline and at Week 24, despite improvements from baseline to Week 24 being observed.

Overall, the studies included in this analysis covered patients from a large range of ethnicities and geographical locations including centres in North America, South America, Europe, Africa, Asia and Australia. However, as is often the case with clinical trials, there was an apparent bias towards Caucasian patients and economically developed Western countries, Although it was beyond the scope of this study, a sub analysis by location and ethnicity would be of interest, although it is likely that numbers of patients in each subcategory may be too low to obtain statistically significant results. Furthermore, how closely these correspond to real-world outcomes is unclear. Previous studies suggest quite large differences between countries with regards to utilization of various treatments, such as lipid-lowering therapies [32, 33]. Higher income countries will tend to have higher coverage of screening and treatment, therefore reducing the burden of disease. Access to disease management programmes has also been shown to help patients achieve greater LDL-C reductions and control rates, which again is likely to result in differences between countries with regards to real-world outcomes [34].

The main strengths of our study are that the data included are patient-level data, and that the population is derived from prospective randomized trials with defined and consistent titration regimens and treatment targets. The pooling of patient data increases statistical power and reduces variability. The main limitation of our study is the age of the data (the most recent of the studies included was conducted in 2008); in addition, statin intensity data was not available. However, viewing this data alongside current recommendations highlights the need for better lipid control in the T2D population [17, 19, 22].

We observed no interaction (or only minor interaction) between glucose-lowering treatment and LDL-C/HDL-C levels. However, there was a tendency of improved effect of lipid-lowering therapy in patients with CVD after 24 weeks of treatment for hyperglycaemia.

In conclusion, this post hoc pooled analysis of randomized controlled trials of people with T2D demonstrates a modest improvement in non-HDL-C and triglyceride levels following antihyperglycaemia therapy in study participants with both T2D and CVD. Despite guideline recommendations, many participants with T2D and CVD did not receive lipid-lowering therapy. In addition, participants had lipid levels outside the optimal range for most categories of lipid control. These data suggest a need for a greater awareness of the risks of CVD in patients with diabetes and for more widespread implementation of guideline recommendations for the management of dyslipidaemia in these individuals.

## Abbreviations

ACC/AHA: American College of Cardiology/American Heart Association
ANCOVA: Analysis of covariance
HbA1c: Glycosylated haemoglobin
BMI: Body mass index
CVD: cardiovascular disease
DET: insulin detemir
FPG: fasting plasma glucose
GLIM: Glimepiride
HDL-C: high-density lipoprotein cholesterol
Gla-100: insulin glargine 100 units/mL
LIS: insulin lispro
LDL-C: low density lipoprotein cholesterol
MET: Metformin
NHANES: National Health and Nutrition Examination Survey
Non-HDL-C: non-high-density lipoprotein cholesterol
OAD: oral antihyperglycaemia drug
PIO: Pioglitazone
ROSR: rosiglitazone
SITA: Sitagliptin
SU: sulfonylurea
TZD: thiazolidinedione
T2D: Type 2 diabetes
US: United States
